# Mitochondrial bioenergetic dysfunction in the D2.*mdx* model of Duchenne muscular dystrophy is associated with microtubule disorganization in skeletal muscle

**DOI:** 10.1371/journal.pone.0237138

**Published:** 2020-10-01

**Authors:** Sofhia V. Ramos, Meghan C. Hughes, Luca J. Delfinis, Catherine A. Bellissimo, Christopher G. R. Perry

**Affiliations:** School of Kinesiology and Health Sciences, Muscle Health Research Centre, York University, Toronto, Ontario, Canada; University of Minnesota Twin Cities, UNITED STATES

## Abstract

In Duchenne muscular dystrophy, a lack of dystrophin leads to extensive muscle weakness and atrophy that is linked to cellular metabolic dysfunction and oxidative stress. This dystrophinopathy results in a loss of tethering between microtubules and the sarcolemma. Microtubules are also believed to regulate mitochondrial bioenergetics potentially by binding the outer mitochondrial membrane voltage dependent anion channel (VDAC) and influencing permeability to ADP/ATP cycling. The objective of this investigation was to determine if a lack of dystrophin causes microtubule disorganization concurrent with mitochondrial dysfunction in skeletal muscle, and whether this relationship is linked to altered binding of tubulin to VDAC. In extensor digitorum longus (EDL) muscle from 4-week old D2.*mdx* mice, microtubule disorganization was observed when probing for α-tubulin. This cytoskeletal disorder was associated with a reduced ability of ADP to stimulate respiration and attenuate H_2_O_2_ emission relative to wildtype controls. However, this was not associated with altered α-tubulin-VDAC2 interactions. These findings reveal that microtubule disorganization in dystrophin-deficient EDL is associated with impaired ADP control of mitochondrial bioenergetics, and suggests that mechanisms alternative to α-tubulin’s regulation of VDAC2 should be examined to understand how cytoskeletal disruption in the absence of dystrophin may cause metabolic dysfunctions in skeletal muscle.

## Introduction

In Duchenne muscular dystrophy, mutations in the X-linked gene dystrophin leads to progressive weakness in striated muscles. Occurring in 1:3500–5000 males, the absence of this cytoskeletal-sarcolemmal linker protein results in a compromised cell membrane that becomes damaged after contraction [1, 2]. While persistent calcium influx has been linked to repeated cycles of fibre degeneration and regeneration [2], the loss of dystrophin has also been shown to cause disorganization of microtubules, specifically measured as α-tubulin, in extensor digitorum longus (EDL) muscle in C57Bl/10*mdx* mice, given dystrophin is a microtubule anchor [3, 4]. However, the manner by which this altered microtubule network contributes to metabolic dysfunction remains unclear.

Mitochondrial dysfunctions have also been identified in limb skeletal muscle, diaphragm and heart from mouse models of Duchenne muscular dystrophy [5–9]. A specific impairment in the ability of ADP to attenuate mitochondrial H_2_O_2_ emission during impaired oxidative phosphorylation was identified in multiple muscles at 4 weeks of age in D2.*mdx* mice [6]. The cause for this mitochondrial dysfunction was not identified, although multiple stressors inherent in the disease could be contributors. One possibility may be that mitochondrial dysfunctions arise from a disorganized cytoskeletal network that was reported previously in the C57Bl/10.*mdx* model [3, 4]. Specifically, as mitochondria are known to bind to tubulin, a component of microtubules [10–13], it is possible that altering microtubule architecture may influence mitochondrial bioenergetics. Indeed, we have previously shown that inducing microtubule disorganization in EDL with microtubule-stabilizing and destabilizing compounds alters ADP’s control of mitochondrial bioenergetics [13]. As such, it seems plausible that the disorganized microtubule network in EDL from C57Bl/10.*mdx* mice [3] are related to mitochondrial dysfunctions observed in other muscles, particularly white gastrocnemius which shares similar fibre type as EDL [5–9]. However, this relationship has not been definitively demonstrated within the same muscle of the same mouse model, nor has a potential mechanism been explored for how microtubules may alter mitochondrial function in this disorder.

The first objective of the present study was to determine if disorganized microtubules are associated with a loss of ADP’s central control of mitochondrial bioenergetics in EDL muscle of D2.*mdx* mice. We have previously used this mouse model to explore mitochondrial dysfunction in other muscles but it has yet to be examined for microtubule disorganization [6, 8]. The second objective was to explore whether this potential relationship was related to altered tubulin-VDAC binding stemming from disorganized microtubules. Specific attention was given to α-tubulin considering it binds various isotypes of β-tubulin as an α/β heterodimer with the CTT tail of both components having affinity for VDAC [10, 14–16]. VDAC2 was selected given 1) its deficiency results in embryonic death thereby demonstrating its importance [17], 2) it has been proposed that VDAC2 may uniquely regulate the more efficient creatine-dependent mitochondrial phosphate shuttling mechanism [18–21], and 3) we have previously shown that tubulin-VDAC2 interactions are changed when microtubule organization is altered by paclitaxel [13]. The results demonstrate a relationship between microtubule disorganization and impaired ADP attenuation of H_2_O_2_ emission. Contrary to the hypothesis, this dysfunction was not associated with altered α-tubulin-VDAC2 binding as detected by a proximity ligation approach (<30nm resolution) [22]. These findings highlight the association between microtubule networks and mitochondrial dysfunction in dystrophin deficiency suggesting an important role of cytoskeletal architecture in mediating metabolic dysfunction in this disease. The results also challenge the model of tubulin regulation of VDAC permeability to ADP, although alternative mechanisms for future investigation are discussed.

## Materials and methods

### Animal care

Briefly, male 4-week old D2.*mdx* mice [23, 24] were used from a colony at York University originally established with breeding pairs from Jackson Laboratories (Bar Harbor, United States). Due to breeding difficulties in the background strain, separate male wildtype (WT) DBA/2J mice were purchased from Jackson Laboratories and were acclimated for 72 hours before experiments were performed. All experiments and procedures were approved by the Animal Care Committee at York University (AUP approval number; 2016–18). Other muscles from these mice were used for separate manuscripts in preparation at the time of this submission.

### Preparation of permeabilized muscle fibre bundles (PmFB)

All experimental procedures were completed as reported previously [6, 13, 25–27]. Mice were anesthetized with 5% isoflurane (1–2 L/min medical air) and maintained at a 3–5% isoflurane for the duration of the tissue harvest. EDL muscles were removed and quickly placed in BIOPS buffer containing (mM): 50 MES, 7.23 K_2_EGTA, 2.77 CaK_2_EGTA, 20 Imidazole, 0.5 Dithiothreitol (DTT), 20 Taurine, 5.77 ATP, 15 phosphocreatine and 6.56 MgCl_2_·6H_2_O (pH 7.1) [6, 13, 25–27] on ice. Tissue was trimmed of fat and connective tissue in BIOPS buffer maintained at 4°C and separated using antimagnetic needle-tipped forceps under magnification (Zeiss 2000, Germany). Each 1–3 mg bundle was permeabilized with 40μg/ml saponin in BIOPS for 30 min. PmFB allocated for pyruvate-induced H_2_O_2_ emission were permeabilized in the presence of 35μM 2,4-dinitrochlorobenzene (CDNB) to remove endogenous glutathione and permit detection of H_2_O_2_ emission supported by the pyruvate dehydrogenase complex [28]. Once permeabilized, PmFB were washed for 15 min at 4°C in MiRO5 buffer containing (mM): 0.5 EGTA, 10 KH_2_PO_4_, 3 MgCl_2_·6 H_2_O, 60 K-lactobionate, 20 hepes, 20 taurine, 110 sucrose and 1 mg/ml fatty acid free BSA (pH 7.1) for respiration experiments, buffer Z containing (mM): 105 K-MES, 30 KCl, 10 KH_2_PO_4_, 5 MgCl_2_·6 H_2_O, 1 EGTA and 5 mg/ml BSA (pH 7.1) for H_2_O_2_ emission assays and buffer Y containing (mM): 250 sucrose, 10 tris-HCl, 20 tris Base, 10 KH_2_PO_4_, and 0.5mg/ml BSA for 10 min and then again in buffer Y with 10μM blebbistatin to determine calcium retention capacity. All wash steps were completed at 4°C.

### Mitochondrial bioenergetic assays

PmFB were placed into a high-resolution respirometer (Oroboros Instruments, Corp. Innsbruck, Austria) in the presence of 20mM creatine to promote cytoplasmic-mitochondrial cycling of creatine/phosphocreatine (“phosphate shuttling”) through mitochondrial creatine kinase (mtCK) in the inner membrane space [19–21, 29]. Approximately 350μM of O_2_ was added to each chamber with each experiment completed before reaching 150μM O_2_. Other technical details of respirometer settings and conditions are described previously [8, 13, 25–27, 30]. Experiments were performed in the presence of 5μM blebbistatin to prevent ADP-induced muscle contraction [25, 31, 32] and normalized to wet weight. State 3 respiration was supported by 5mM pyruvate + 2mM malate (NADH, complex I) followed by ADP titrations at 25, 100, 500 and 5000μM ADP. 10μM cytochrome *c* was added to test for intactness of the outer mitochondrial membrane, with all responses exhibiting <15% increase in respiration. 20mM succinate (FADH_2_) was then added for complex II-supported respiration.

Separate PmFB were placed into a quartz cuvette containing 1ml of Buffer Z containing 10μM Amplex UltraRed, 0.5U/ml horseradish peroxidase, 40U/ml Cu/Zn-SOD1, 1 mM EGTA, 20mM creatine and 5μM blebbistatin to measure H_2_O_2_ emission. Experiments were completed by spectrofluorometry (QuantaMaster 40, HORIBA Scientific, Edison, NJ, USA) with continuous stirring at 37°C. Pyruvate (10mM) and malate (4mM) were used to stimulate mitochondrial H_2_O_2_ emission at complex I (NADH) followed by ADP titrations at 25, 100 and 500μM to attenuate H_2_O_2_ emission. Upon completion, bundles were blotted dry and lyophilized to obtain a dry weight for normalization as previously described [6, 8, 13, 31]. The rate of H_2_O_2_ emission (pmol·s^-1^·mg dry weight^-1^) was then calculated from the slope (F/min) applied to a standard curve established with the same reaction conditions. H_2_O_2_ emission data at each ADP concentration was then divided by the initial maximal rate of H_2_O_2_ emission measured with pyruvate/malate before ADP was added. In so doing, the data captures the physiological importance of ADP in attenuating mitochondrial H_2_O_2_ emission as an index of mitochondrial ‘ADP sensitivity’.

Calcium retention capacity (CRC) was performed s by spectrofluorometry (QuantaMaster 80, HORIBA Scientific, Edison, NJ, USA) with separate PmFB in a Calcium Retention Capacity buffer as previously described [6, 8, 33, 34] with the addition of 5mM ADP to capture the potential effect of ADP on membrane potential as might occur under state 3 conditions *in vivo*. PmFB were placed into a quartz cuvette containing Calcium Green-5N (Invitrogen) dissolved in Buffer Y [33] where an initial 8nm CaCl_2_ pulse was added followed by 4nm CaCl_2_ pulses until mitochondrial permeability transition pore opening was evident. Two 0.5mM pulses of CaCl_2_ were then added to establish maximum fluorescence by saturating the fluorophore. Similarly to H_2_O_2_ bundles, PmFB were lyophilized to obtain dry weights for normalization.

### Single fibre isolation, immunofluorescent staining and proximity ligation assay

A separate set of D2.*mdx* and control DBJ/2J mice (*n* = 8–10) were used for immunofluorescent experiments as described previously [13]. Briefly, EDL single fibres were isolated with 0.2% collagenase type 4 (Worthington, LLS004188, Lakewood, NJ) for 70 min (maintained at 37°C) and triturated until viable single fibres were released. Fibres were fixed with 4% paraformaldehyde for 10 min, permeabilized with 0.01% triton-X100 for 10 min and then blocked with 5% BSA PBS^++^ for 60 min, all at room temperature. Samples were then incubated with α-tubulin (1:1000 Sigma, T6199) for 4 hours at room temperature followed by VDAC2 overnight at 4°C (1:250, Santa Cruz, 32059). Half of the fibres were used for detection of α-tubulin following incubation with the secondary antibody Alexa Flour 488 (Invitrogen, A21121).

The remaining fibres retained following primary antibody incubations were used for determination of protein-protein interaction by proximity ligation assay. Fibres were probed according to manufacturer’s instructions with some modifications described previously [13]. Briefly, proximity ligation assay anti-goat minus (Sigma, DUO92001) and anti-mouse plus (Sigma, DUO92006) probes were used to detect primary antibodies used above. Single fibres were incubated with anti-goat minus and anti-mouse plus probes (1:5 dilution) for 1 hour prior to a treatment with the detection reagents red (Sigma, DUO92008) consisting of a 30 min incubation with the provided ligase to splice the oligonucleotide ends of the probes together, and a 1 hour 40 min incubation with the provided polymerase to read and amplify the signal on the resulting DNA strand. All incubations were completed at 37°C. Fibres were coated in anti-fade mounting medium and covered with a coverslip. Negative control experiments were previously published that demonstrate the specificity of the proximity ligation assay with these same antibodies [13].

### Image capture and quantitation

**Fig 1:**
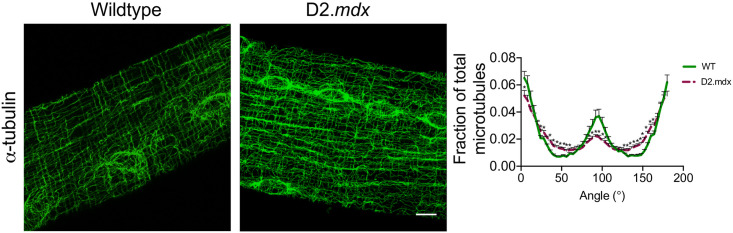
Microtubule organization. Confocal microscopy representative 3D images of α-tubulin-stained single EDL fibres from WT (9 slices) and D2.*mdx* (10 slices) mice. Graphical representation of microtubule directionality measured with the TeDT software (*n* = 5–8). Results are reported as mean ± SEM, (*p<0.05 vs wildtype). Scale bar, 10μm.

A Zeiss laser scanning confocal microscope 700 (Carl Zeiss) was used to acquire images with a 63X oil immersion objective with the pin hole set to 1AU. The parameters used to acquire images of microtubules include; excitation at 488nm for α-tubulin obtaining an average intensity for each image. Starting at the top of the fibre, capturing 4–19 stacks with a z-step of 0.35 μm resulting with a depth of approximately 3.94 ± 0.11 μm^2^ suggesting that predominately sub-sarcolemma microtubules were imaged for both directionality and protein-protein interaction analysis. Image acquisition began when microtubules were clearly visible. Along the length of the fibre, 3 different images were taken, scanning the same area (μm^2^) in each image (yielding 3 images/fibre) with one fibre analyzed per mouse (WT and D2.*mdx*). The representative image used in this manuscript (Fig 1) was stacked into a 3D image using ImageJ (ImageJ, http://imagej.nih.gov/ij/) projecting an average intensity. Samples were excited at 594nm to capture α-tubulin-VDAC2 interactions completed with the proximity ligation assay. Similar parameters were used to acquire α-tubulin-VDAC2 interactions. Images were analyzed in 3D using the spot tool on Imaris image quantifying software (Bitplane).

### Microtubule directionality

Confocal images of microtubules were utilized for the determination of microtubule directionality as described previously [35]. Briefly, images were prepared by selecting 6 ROI of 100x100 square pixels for each image obtained, not including areas around nuclei. A single z-stack image obtained at approximately 4–6 stacks from the top of the fibre were utilized for directionality analysis. Images were processed using the sharpen and smooth tool on ImageJ. Microtubule organization was then assessed using the TeDT software kindly provided by Dr. Evelyn Ralston at the National Institute of Arthritis and Musculoskeletal and Skin Disease at the National Institute of Health (NIAMS at NIH), Bethesda, Maryland, USA. The plotted graph represents the average frequency of each angle that microtubules are oriented. Results are expressed as fractions of total microtubules aligned at each degree.

### Western blotting

Western blotting procedures were completed as previously reported [6, 8, 36] using rodent OXPHOS Cocktail, ab110411, Abcam, Cambridge, UK, 1:250 dilution), VDAC2 (Santa-Cruz, 32059, Dallas, TX, 1:1000 dilution) and adenine nucleotide translocase 1 (ANT1, ab180715, Abcam, 1:1000 dilution) antibodies. Briefly, protein content was determined using BCA protein assay kit (Life Technologies, Carlsbad, CA, USA) to prepare samples. Proteins were separated on a 12% acrylamide gel and then transferred onto a low-fluorescence polyvinylidene difluoride membrane that was then blocked and incubated with the respective primary antibodies listed above overnight at 4°C. Membranes were then washed and incubated with their corresponding infrared fluorescent secondary antibodies (LI-COR, Lincoln, NE, USA) and imaged with the LI-COR infrared imager. Membranes were analysed with ImageJ software. Proteins were made relative to total protein measured on a separate membrane stained with amido black stain where loading accuracy was tested with a coefficient of variation of 8.6%.

### Statistics

The ROUT test was used to omit outliers followed by the D’Agostino-Pearson omnibus normality test to verify that all data followed a normal distribution. Statistical differences were assessed by two-way ANOVA for ADP-stimulated respiration and attenuation of H_2_O_2_ emission followed by Bonferroni multiple comparison post-hoc analysis when appropriate. Differences in glutamate and succinate stimulated respiration, H_2_O_2_ emission in the absence of ADP, Western blot densitometry results and CRC were determined through a student’s un-paired t-test (GraphPad Prism 7, La Jolla, CA). Similarly, a student’s un-paired t-test was used to determine differences in the average frequency calculated at each angle when measuring microtubule directionality. Results are reported as mean ± SEM with significance accepted at *p*< 0.05.

## Results

### Altered microtubule organization is associated with impaired ADP-control of bioenergetics in D2.*mdx* mice

**Fig 2:**
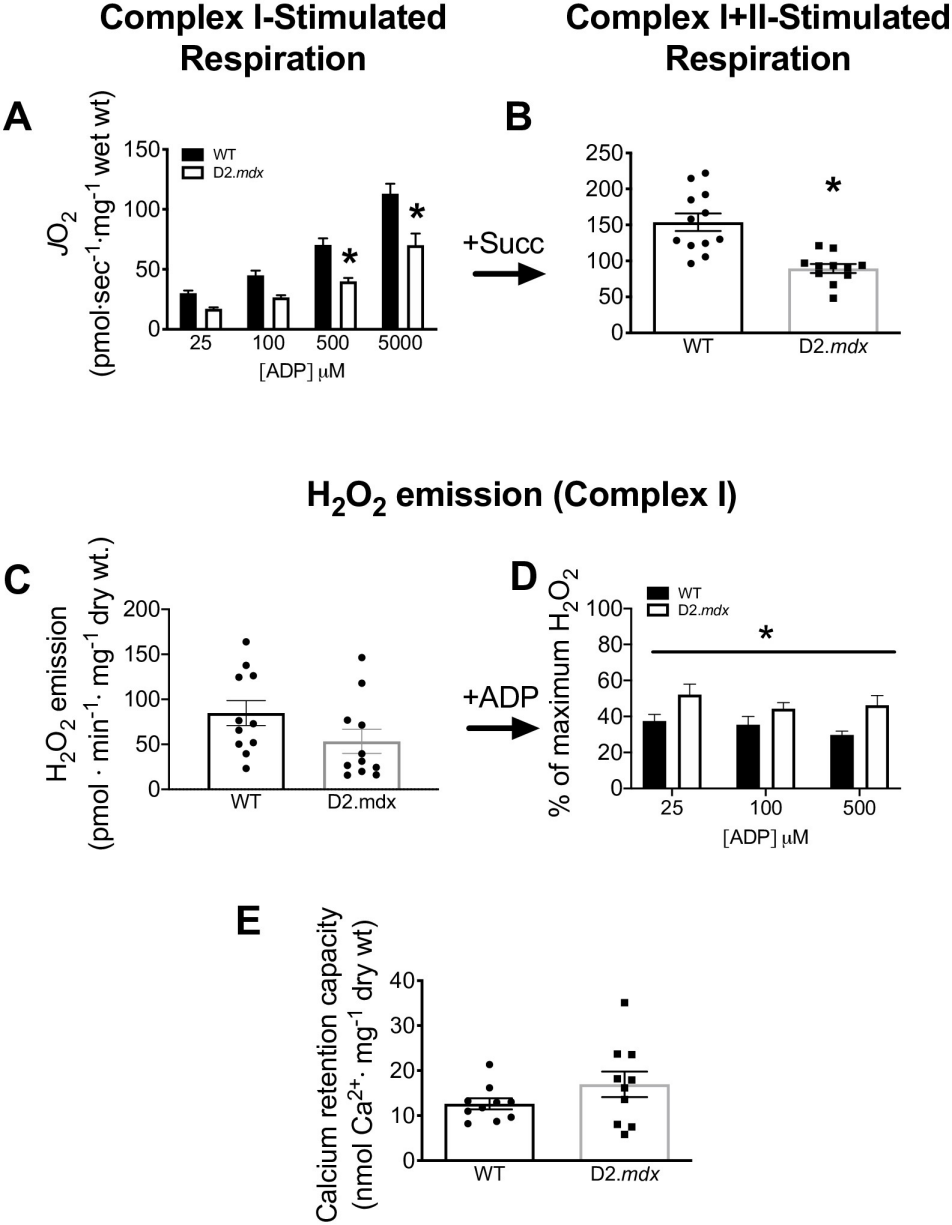
Mitochondrial bioenergetics in EDL PmFB. ADP-stimulated respiration was initially supported by complex I (NADH from 5mM pyruvate/4mM malate) in the presence of creatine with an ADP titration of 25μM, 100μM, 500μM and 5mM concentrations (*n* = 12) **(A)** directly followed by complex II (FADH_2_ from 20mM succinate, Succ) (*n* = 11–12) **(B)**. Mitochondrial H_2_O_2_ emission was stimulated at complex I (NADH from 10mM pyruvate/4mM malate) (*n* = 12) **(C)** followed by an ADP titration and expressed relative to maximal H_2_O_2_ emission in response to pyruvate/malate before ADP was titrated (% of State II) (*n* = 11–12) **(D)**. Calcium retention capacity (*n* = 10) **(E)**. Results are reported as mean ± SEM, (*p<0.05 vs wildtype).

Confocal microscopy visually confirmed the disorganization of microtubule architecture in EDL from D2.*mdx* compared to WT as was reported previously in C57.Bl/10*mdx* (Fig 1) [37, 38]. Further analysis with the TeDT software determined a higher frequency of microtubules oriented at various direction from D2.*mdx* mice when compared to WT which had higher peaks at 0/180 and 90 degrees (Fig 1). As microtubules have been proposed to regulate ADP permeability through VDAC, we next determined the ability of ADP to stimulate respiration and lower H_2_O_2_ emission. Complex I-supported respiration (NADH from pyruvate) was impaired in D2.*mdx* vs WT at 500μM (-43%, *p*<0.001) and 5mM ADP (-38%, *p*<0.0001) with a main effect observed across groups (p<0.0001) (Fig 2A), as was combined complex I and II (additional FADH_2_ from succinate) (*p*<0.001) (Fig 2B).

Maximal H_2_O_2_ emission (State II, no ADP) was similar in D2.*mdx* and WT (*p* = 0.11) (Fig 2C). The ability of ADP to attenuate H_2_O_2_ was impaired in the D2.*mdx* mice when compared to WT at all ADP concentrations that were assessed (main effect p<0.05) (Fig 2D). This impairment in ADP was not observed in the absence of creatine in the media (data not shown). While ADP sensitivity is captured here by examining the change in H_2_O_2_ emission relative to maximal State II conditions, no change in absolute rates of H_2_O_2_ emission were observed at any given ADP in EDL (data not shown).

We next employed a calcium retention capacity assay to determine whether dystrophic muscle is more susceptible to mPTP formation (which is believed to involve VDAC) [39]. However, no differences were observed between WT and D2.*mdx* EDL (*p* = 0.17) (Fig 2E).

### Reduced ANT1 protein content, but not α-tubulin-VDAC2 interactions, may contribute to mitochondrial ADP-impairments in D2.*mdx* mice

**Fig 3:**
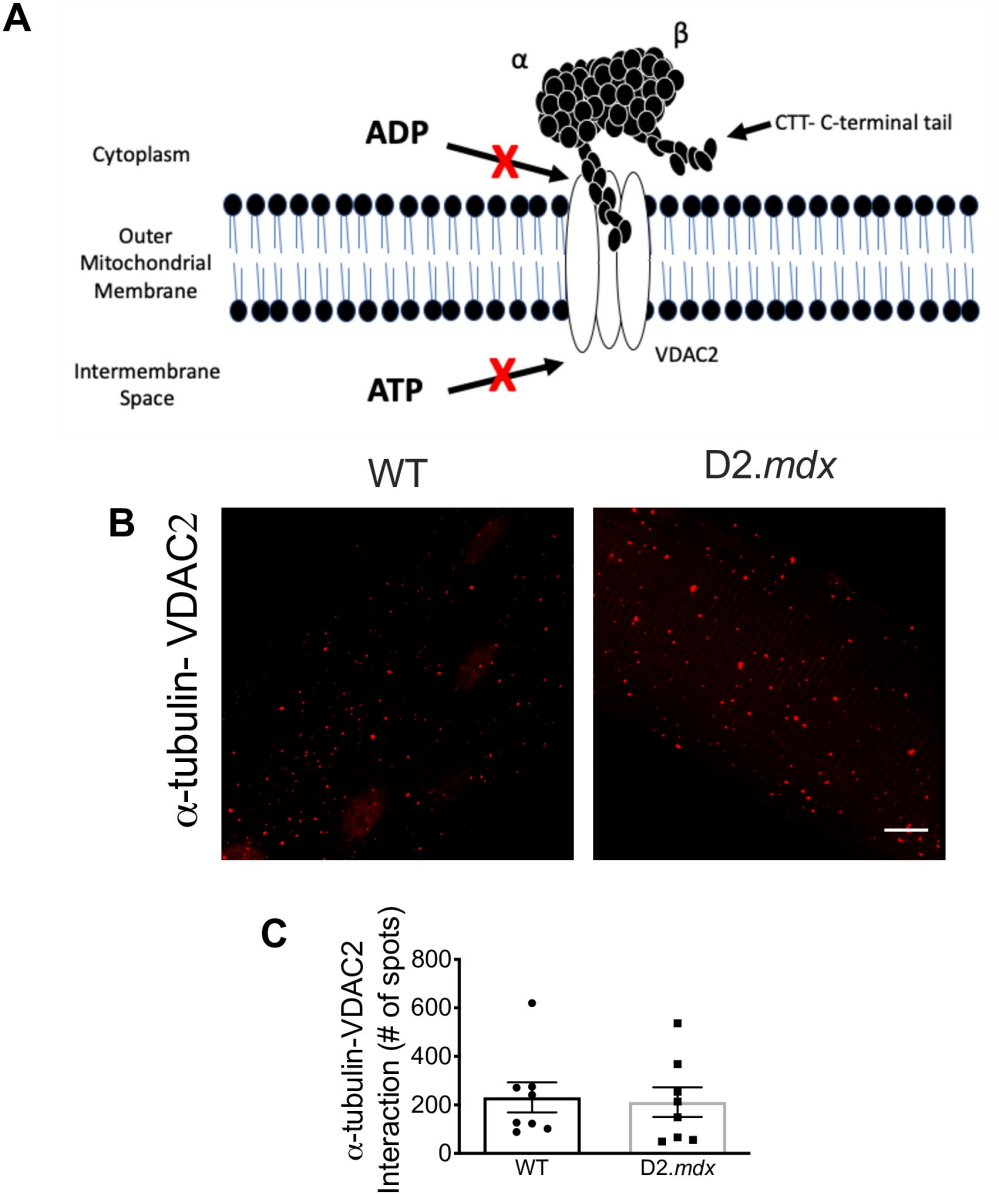
α-tubulin—VDAC2 interactions in single EDL fibres. Schematic representing the model of α-tubulin-VDAC2 interaction **(A)**. Cropped confocal microscopy images **(B**; see raw data for full representative image**)** and graphical depiction of the proximity ligation assay of α-tubulin-VDAC2 (*n* = 8) **(C)**. Scale bar, 10μm. Results are reported as mean ± SEM. The image in panel A is reproduced with permission from our previous work [13].

**Fig 4:**
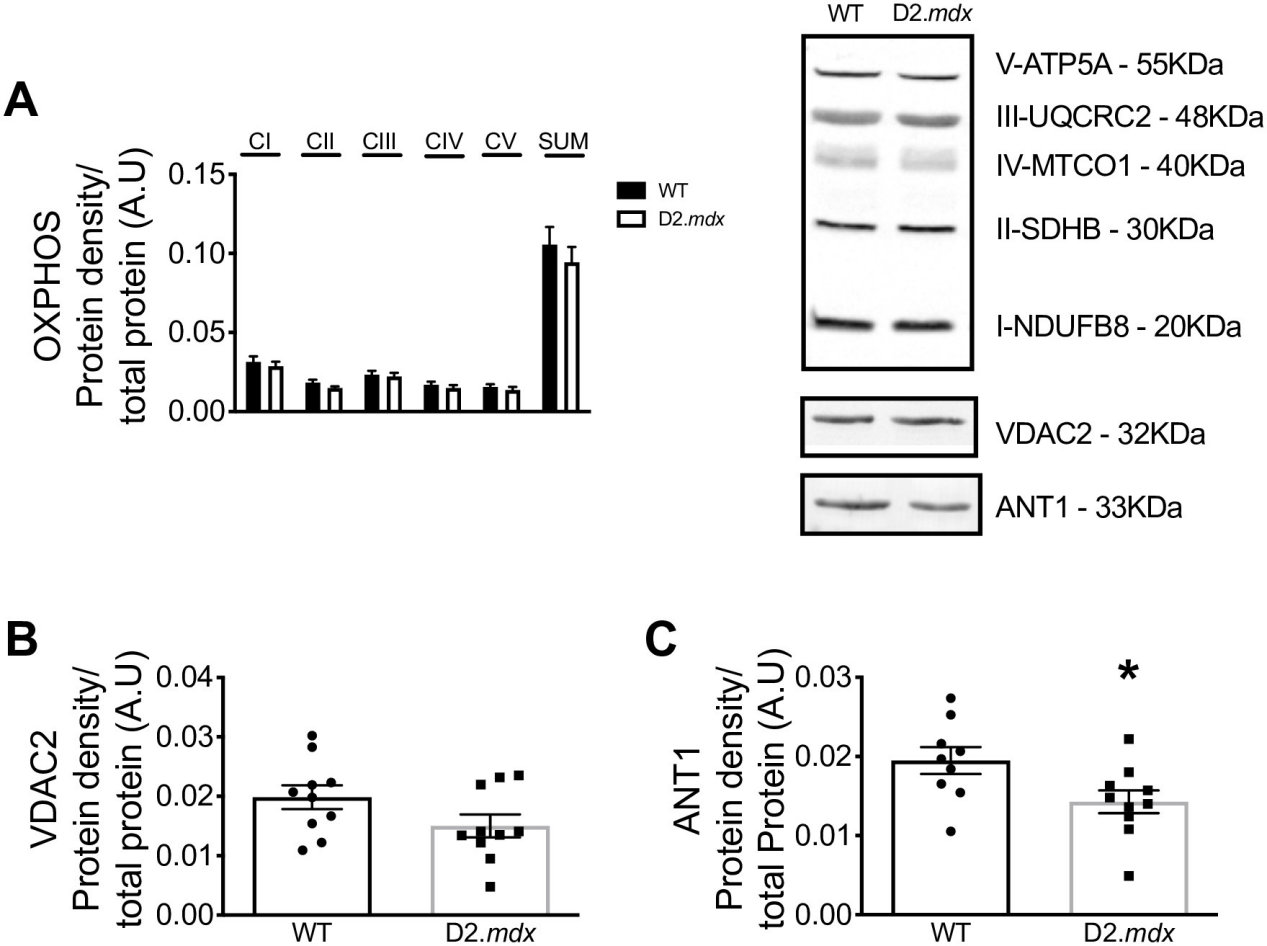
Protein content of mitochondrial proteins. Integrated densitometry for subunits of electron transport chain complexes I-V (OXPHOS; *n* = 10–11), **(A)** VDAC2 (*n* = 10) **(B)** and ANT1 (*n* = 9–10) **(C)** with representative blots. Results are reported as mean ± SEM, (*p<0.05 vs wildtype).

A proximity ligation assay was used to determine whether α-tubulin-VDAC2 interactions were different between D2.*mdx* and WT [10, 13, 40]. However, similar protein-protein interactions were found in D2.*mdx* and WT mice (*p* = 0.83) (Fig 3). No changes were observed in specific subunits of complexes I (*p* = 0.54), II (*p* = 0.12), III (*p* = 0.70), IV (*p* = 0.50) or V (*p* = 0.48) nor their sum (*p* = 0.45) (Fig 4A). VDAC2 (*p* = 0.10) (Fig 4B) protein content was similar between WT and D2.*mdx* while the inner mitochondrial membrane transport protein adenine nucleotide translocase 1 (ANT1) was significantly reduced in D2.*mdx* compared to WT (-27%, *p*<0.05) (Fig 4C).

## Discussion

In D2.*mdx* mice, we demonstrate that microtubule disorganization in EDL muscle is associated with a reduced ability of ADP to stimulate mitochondrial oxidative phosphorylation and attenuate H_2_O_2_ emission. However, contrary to the hypothesis, there were no differences in the degree of α-tubulin-VDAC2 interactions assessed by proximity ligation assay. These findings demonstrate that microtubule disorganization and mitochondrial dysfunction occur concurrently in dystrophin-deficient muscle, but that the tubulin-VDAC model of bioenergetic control may not represent a causal link between these two phenomena leaving the possibility that other mechanisms may exist.

Impaired mitochondrial bioenergetics is thought to contribute to muscle weakness in Duchenne muscular dystrophy [6–8, 41]. However, the specific link between dystrophin deficiency and mitochondrial dysfunction has not been fully resolved. While Ca^2+^ stress has been proposed as a primary cause of swollen mitochondria and impaired oxidative phosphorylation in *mdx* mice [5], separate observations of altered microtubule networks [37, 38, 42] were also linked to disrupted cytosolic NADPH oxidase-induced ROS and Ca^2+^ signaling [42]. An intriguing possibility of a mitochondrial link to microtubule disorganization emerges when considering the separate discoveries that tubulin components of microtubules can directly bind to VDAC on the outer mitochondrial membrane and decrease its permeability to ADP/ATP cycling [10, 14, 21, 29]. Considering that pharmacological alterations of microtubule networks changes tubulin-VDAC interactions and ADP-dependent bioenergetics [13], it seems plausible that the distinct observations of disorganized microtubules and mitochondrial dysfunctions in D2.*mdx* muscle may be due to altered microtubule-VDAC interactions. Such an observation would implicate microtubule disorganization in D2.*mdx* mice as a modulator of mitochondrial dysfunction in addition to the cytosolic stressors noted previously [42].

However, the present findings demonstrate no differences in the degree of α-tubulin-VDAC2 interactions despite the association between microtubule disorganization and mitochondrial dysfunction. Nevertheless, this observation does not rule out the possibility of other tubulin-VDAC interaction combinations. For example, while the CTT tail of α-tubulin has been shown to block the pore of VDAC, there is a greater affinity of the CTT tail on certain β isotypes, particularly βIII and VDAC1 [14]. In addition, it has been suggested that free βII tubulin binds VDAC in muscle—independent of heterodimeric tubulin—such that this free pool may be a distinct regulator of ADP/ATP cycling through VDAC [15]. Furthermore, the affinity of tubulin binding can be modulated through post translational modifications [11] such as phosphorylation [43] or altered mitochondrial membrane lipid composition [44] which highlights the complexity of the potential regulation of this pathway. Lastly, it has been proposed that free tubulin regulates VDAC permeability based on experiments that observed the effect of adding or removing exogenous free tubulin to various preparations [10, 16]. Future investigations could develop novel approaches that capture the degree to which free tubulin binds VDAC without altering their interactions during specimen processing. Such approaches would be required to isolate the relative contribution of free vs. polymerized tubulin to VDAC-dependent bioenergetics in the absence of dystrophin. Overall, the present findings warrant additional investigation into these alternative possibilities of how microtubule network dynamics may change the regulation of ADP-control of bioenergetics through VDAC.

This study does not rule out the possibility that VDAC1 or VDAC3 are differentially affected by disorganized microtubule networks in D2.*mdx* muscle. However, it has been suggested that only VDAC2 regulates the effect of creatine on respiration through phosphate shuttling [18], as assessed in the present study, possibly by being functionally linked to mitochondrial creatine kinase as well as tubulin in a super-complex [29]. The lethality of VDAC2 knockout mice also suggests its importance in regulating mitochondrial bioenergetics [17]. Nevertheless, the lack of change in α-tubulin-VDAC2 in the present investigation warrants further study of VDAC1 and 3 interactions with tubulin isotypes in *mdx* muscle given their influence on respiration [17].

Lastly, an additional relationship was found between reduced ANT1 content and impaired ADP-dependent mitochondrial bioenergetics. As ANT1 is found on the inner mitochondrial membrane, it is not thought to bind tubulin directly but may still be part of a larger complex with mitochondrial creatine kinase and VDAC [21]. Reduced ANT1 may be a distinct contributor to impaired ADP-control of bioenergetics in EDL muscle similar to the reductions previously reported in white gastrocnemius and quadriceps in D2.*mdx* mice at the same age of 4 weeks [6, 8].

In conclusion, this investigation demonstrates that microtubule disorganization is associated with mitochondrial dysfunction within the same muscle of dystrophin-deficient mice, but this may not be mediated by altered α-tubulin-VDAC2 interactions. Additional research is warranted given the proposed model of tubulin-VDAC regulation of bioenergetics is complex and may involve other factors such as alternative tubulin and VDAC isoforms. The association between microtubule organization and mitochondrial dysfunction reported herein serves as a foundation for extensive exploration between these various combinations to determine if microtubules truly ‘link’ dystrophin deficiency to mitochondrial bioenergetics.

## Supporting information

S1 Raw images (DOCX)

S1 Raw data (XLSX)
