# Supplementary material for: Mitochondrial bioenergetic dysfunction in the D2.mdx model of Duchenne muscular dystrophy is associated with microtubule disorganization in skeletal muscle
Source: PLoS One. 2020 Oct 1;15(10):e0237138. doi: 10.1371/journal.pone.0237138 (PMC7529311; doi:10.1371/journal.pone.0237138)
Supplement: S1 Raw images — (DOCX) [file pone.0237138.s001.docx]

PONE-D-19-09897R4 raw images detected with LI-COR Odyssey infrared imager.

Figure 4A. OXPHOS.

WT1 mdx1 WT2 mdx3 WT4 mdx4 WT4 mdx5


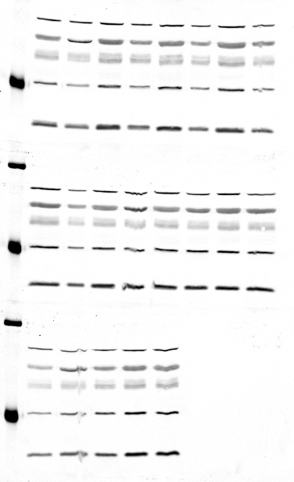


25 kDa

WT6 mdx6 WT8 mdx7 WT9 mdx8 WT10 mdx9


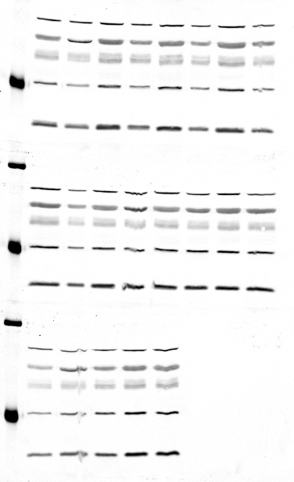


75 kDa

25 kDa

WT12 mdx10 WT13 mdx11 and 12


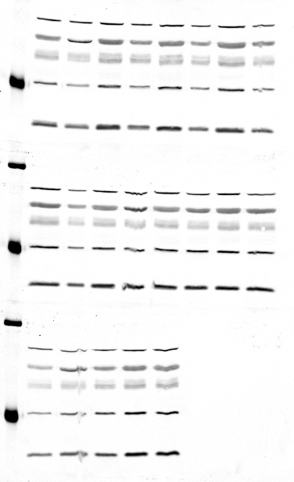


75 kDa

25 kDa

Figure 4B. VDAC2. Arrow indicates band that was quantified.


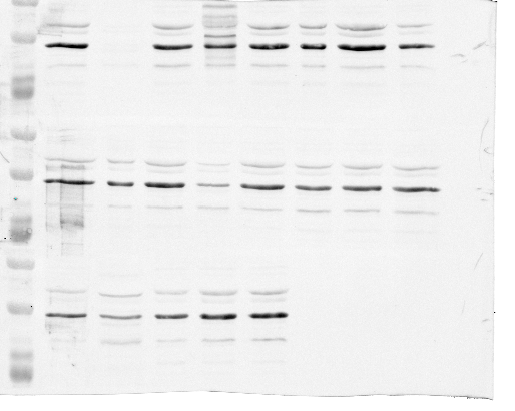


WT1

mdx3

WT2

WT3

WT4

mdx4

mdx5

mdx6

WT8

mdx8

mdx9

mdx10

mdx11 and 12

WT6

mdx7

WT9

WT10

WT12

WT13

mdx 1

50kDa

37kDa

50kDa

37kDa

50kDa

37kDa

25kDa

20kDa

25kDa

20kDa

25kDa

20kDa

Figure 4C. ANT1

Red circle was dropped.


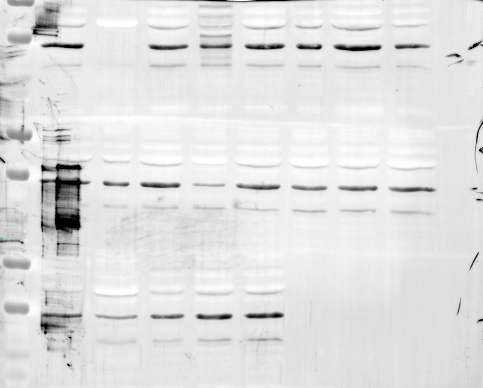


WT1

mdx3

WT2

WT3

WT4

mdx4

mdx5

mdx6

WT8

mdx8

mdx9

mdx10

mdx11 and 12

WT6

mdx7

WT9

WT10

WT12

WT13

mdx1

50kDa

37kDa

50kDa

37kDa

50kDa

37kDa

Loading Control.


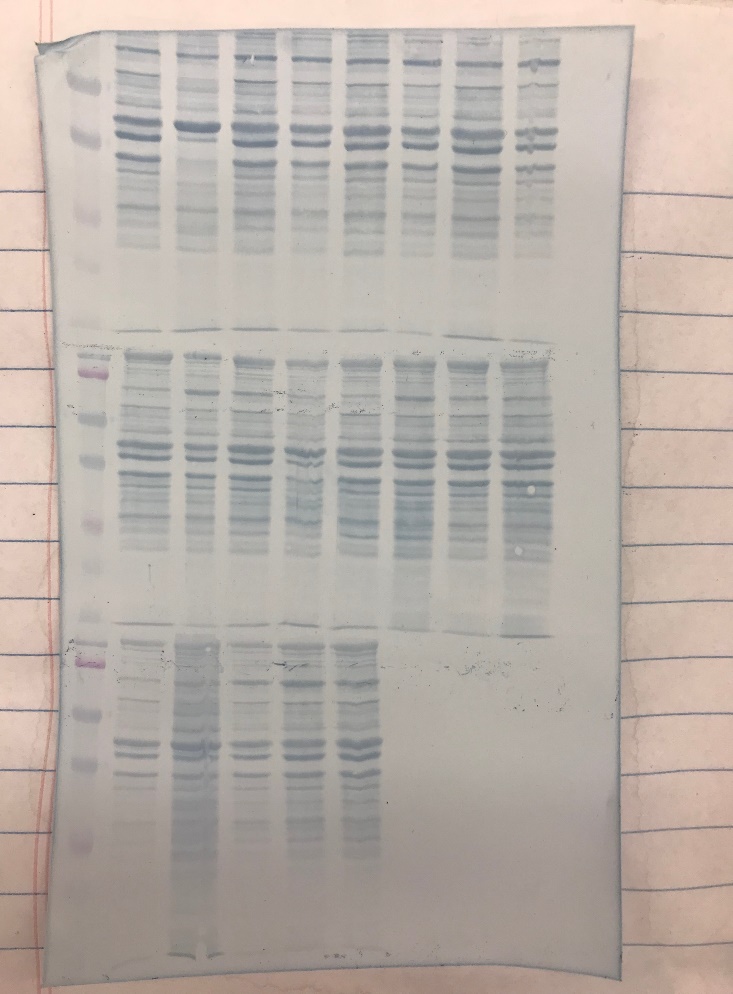


WT1 mdx1 WT2 mdx3 WT3 mdx4 WT4 mdx5

WT6 mdx6 WT8 mdx7 WT9 mdx8 WT10 mdx9

WT12 mdx10 WT13 mdx11 and 12

50kDa

37kDa

50kDa

37kDa

50kDa

37kDa

25kDa

20kDa

25kDa

20kDa

75 kDa

75 kDa
